# Genomic Insight into Zoonotic and Environmental *Vibrio vulnificus*: Strains with T3SS2 as a Novel Threat to Public Health

**DOI:** 10.3390/microorganisms12112375

**Published:** 2024-11-20

**Authors:** Ling-Chao Ma, Min Li, Yi-Ming Chen, Wei-Ye Chen, Yi-Wen Chen, Zi-Le Cheng, Yong-Zhang Zhu, Yan Zhang, Xiao-Kui Guo, Chang Liu

**Affiliations:** 1 School of Global Health, Chinese Center for Tropical Diseases Research, Shanghai Jiao Tong University School of Medicine, Shanghai 200025, China; malingchao@sjtu.edu.cn (L.-C.M.); minli@shsmu.edu.cn (M.L.);; 2 School of Public Health, Shanghai Jiao Tong University School of Medicine, Shanghai 200025, China; 3 Department of Immunology and Microbiology, Shanghai Jiao Tong University School of Medicine, Shanghai 200025, China

**Keywords:** *Vibrio vulnificus*, genotype, antibiotic resistance genes (ARGs), virulence, the type III secretion system (T3SS)

## Abstract

*Vibrio vulnificus* is a significant opportunistic pathogen with the highest fatality rate among foodborne microbes. However, due to a lack of comprehensive surveillance, the characteristics of isolates in China remain poorly understood. This study analyzed 60 strains of *V. vulnificus* isolated from diverse sources in Shanghai, including shellfish, crabs, shrimps, throat swabs of migratory birds, as well as seafood farming water and seawater. Identification of the genotypes was performed using PCR, and cytotoxicity was determined using an LDH assay. DNA was sequenced using Illumina NovaSeq followed by a bioinformatic analysis. The results demonstrated that a majority of the strains belonged to the 16S rRNA B-*vcg*C genotype. All strains carried five antibiotic resistance genes (ARGs), with some strains carrying over ten ARGs, mediating resistance to multiple antibiotics. Five strains possessed a highly abundant effector delivery system, which further investigations revealed to be a type III secretion system II (T3SS2), marking the first description of T3SS2 in *V. vulnificus*. Phylogenetic analysis indicated that it belonged to a different genetic lineage from T3SS2α and T3SS2β of *V. parahaemolyticus*. Bacteria with T3SS2 sequences were concentrated in coastal areas and mostly within the genus *Vibrio* in the global prevalence survey. Our study provides essential baseline information for non-clinical *V. vulnificus* and discovers the existence of T3SS2 in several strains which may be more virulent, thereby posing a new threat to human health.

## 1. Introduction

*V. vulnificus* is an important opportunistic pathogen that can cause wound infections and sepsis by wound exposure to seawater or by consuming seafood containing bacteria [1,2]. It is associated with the highest fatality rate among foodborne pathogens [3,4,5,6]. A notable characteristic of infection is the short incubation period, with symptoms typically manifesting within 24 h after exposure [7,8].

To date, various types of *V. vulnificus* have been defined [7]. Initially, it was categorized into the following three biotypes based on biochemical, serological characteristics, and host range [9,10,11]: almost all clinical infections are biotype 1 [4]; biotype 2 can cause severe infections in eels [12,13,14,15], but it rarely leads to human infections [16]; in 1996, biotype 3 emerged among individuals who consumed pond-cultivated fish, leading to a widespread *V. vulnificus* infection that impacted multiple regions throughout Israel [10], and it is considered a chimera of biotype 1 and biotype 2 [17]. Further genetic classifications, such as 16S rRNA typing (type A, B, and AB) [18] and *vcg* typing (*vcg*C and *vcg*E) [19,20], have been used to distinguish between the environmental and clinical strains, with the 16S rRNA B-*vcg*C genotype strains being assumed to be more virulent [21] and regarded as potentially lethal in clinical settings [22,23]. More recent phylogenetic analyses have further divided *V. vulnificus* into multiple lineages, enhancing our understanding of its diversity and pathogenicity [24,25].

The virulence factors (VFs) of *V. vulnificus* encompass acid neutralization, capsular polysaccharide, iron acquisition, motility, adhesion-related proteins, and a range of exotoxins (including the contact-dependent killing toxin MARTX_Vv_ (RtxA1) [26,27,28], phospholipase PlpA [29], cytolysin/hemolysin VvhA [30,31], and elastolytic protease VvpE [32]). Additionally, three complete secretion systems (types I, II, and VI) [33,34,35] have been identified. The multifunctional autoprocessing repeats-in-toxin (MARTX_Vv_/RtxA1), encoded by the *rtxA1* gene, is a key exotoxin exhibiting cytotoxic activity. It is a large single polypeptide comprising multiple effector domains situated between conserved N- and C-terminal repeat sequences [27]. PlpA is capable of necrotic cell death in epithelial cells and lysing human red blood cells [29]. VvhA, a pore-forming toxin, confers strong hemolytic activity to *V. vulnificus* by triggering apoptosis, necrotic cell death, and autophagy through the dysregulation of host cell signaling, resulting in host tissue damage [36]. Furthermore, the *vvhA* gene serves as a specific detection marker for *V. vulnificus* [37]. Elastase VvpE, an extracellular zinc metalloprotease, possesses multiple proteolytic activities that can lead to host tissue damage and inflammation [38]. Type I secretion system (T1SS) regulates the secretion of MARTX_Vv_/RtxA1, while Type II secretion system (T2SS) governs the secretion of VvhA and VvpE [34]. In 2016, Church first found the presence of two types of Type VI secretion systems (T6SS1 and T6SS2) in *V. vulnificus* [35], which play significant roles in bacterial virulence and population dynamics [39].

The type III secretion system (T3SS) is a complex nanomachine that enables bacteria to transfer protein effectors across eukaryotic cell membranes [33]. It was first discovered in *V. parahaemolyticus*, which has two types of T3SS (T3SS1 and T3SS2) [40]. T3SS plays a vital role in the infection process of numerous pathogens [41,42,43]. Based on differences in structural components and amino acid sequences, T3SS2 is divided into three different lineages, namely T3SS2α, T3SS2β, and T3SS2γ [44,45,46]. T3SS2 is involved in enterotoxicity [47] and is present in Kanagawa phenomenon (kp)-positive strains but absent in kp-negative strains [48]. T3SS genes were also found in other *Vibrios* such as *V. cholerae*, *V. alginolyticus*, *V. harveyi*, *V. mimicus*, etc. [44,45,49,50]. In 2023, Sebastian A. Jerez et al. identified putative T3SS2 gene clusters from 1130 bacterial genomes across eight bacterial genera, five bacterial families, and 47 species [51]. This indicates that T3SS2 extends beyond the Vibrionaceae family, and different effector protein libraries may have different impacts on the pathogenic potential and environmental adaptability of each bacterium that acquires the T3SS2 gene cluster.

In this study, we isolated 60 strains of *V. vulnificus* from various sources in Shanghai, China, and conducted a series of investigations on the typing, carriage of ARGs, and virulence genes of these strains. Notably, the T3SS2 gene cluster was found on chromosome 1 of five *V. vulnificus* strains. Mobile genetic elements were identified on both sides of this gene cluster, indicating the occurrence of horizontal gene transfer events. This study also marks the first report of the T3SS2 gene cluster in *V. vulnificus*.

## 2. Materials and Methods

### 2.1. Sample Collection and Characterization

From June to December 2023, a total of 328 samples were collected in Shanghai, China, involving shellfish, crabs, shrimps, throat swabs of migratory birds, as well as seafood farming water and seawater. A total of 60 strains of *V. vulnificus* were isolated from these samples. The strain isolation steps can be summarized as follows: The samples were placed in a peptone–sodium chloride–cellobiose–polymyxin E enrichment broth (PNCC; Hopbiol, Qingdao, China) and incubated at 37 °C for 12 h. Then, the enrichment broth was streaked on cellobiose–polymyxin E agar plates (CC; Hopbiol, Qingdao, China) and cultured overnight at 37 °C. The suspected colonies were initially identified by matrix-assisted laser desorption ionization time-of-flight mass spectrometry (MALDI-TOF MS), and then the *V. vulnificus* hemolysin gene (*vvhA*) was amplified by polymerase chain reaction (PCR) to confirm the isolates [52]. A positive control (*V. vulnificus* ATCC 27562) and a negative control (enzyme-free water) were used. Lastly, all strains after second-generation sequencing had an ANI of ≥95% compared with *V. vulnificus* ATCC27562 by FastANI comparison [53]. All strains were stored at −80 °C in Tryptic Soy Broth (TSB) with 3% NaCl and 20% (vol/vol) glycerol.

### 2.2. Genotyping and Phylogenetic Analysis

Strain typing was performed by PCR to determine the genotype and biotype of each strain, which belonged to the following: 16S rRNA A/B [54], *vcg*C/E [19], *Bt2*, and *SerE* [55]. Each 20 μL reaction for PCR amplification contained the following components: 10 μL of 2 × Pfu PCR Mastermix (TIANGEN, Beijing, China), 1 μL of 10 μM forward primer, 1 μL of 10 μM reverse primer, 6 μL of ddH_2_O, and 2 μL of extracted DNA. Specifically, the following respective primers were used: *vcg*C-F (AGCTGCCGATAGCGATCT), *vcg*C-R (TGAGCTAACGCGAGTAGTGAG), *vcg*E-F (CTCAGAAAGGCTCAATTGAC), *vcg*E-R (GATTAACGCTGTAAGGCCG), 16S rRNA A-F (CATGATAGCTTCGGCTCAA), 16S rRNA A-R (CACTACCACCTTCCTCACGAC), 16S rRNA B-F (GCCTACGGGCCAAAGAGG), 16S rRNA B-R (CCTGCGTCTCCGCTGGCT), *SerE*-F (TGTTGTTCTTGCCCACTCTC), *SerE*-R (CGCGCTTAGATTTCTCTCACC), *Bt2*-F (AGAGATGGAAGAAACAGGCG), *Bt2*-R (GGACAGATATAAGGGCAAATGG).

The cycle conditions were consistent with those reported previously [19,54]. The genotypes of all strains were also confirmed by BLAST comparison with the corresponding genes using their whole genome information.

To compare the evolutionary relationships, we downloaded 60 strains of *V. vulnificus* from different sources, geographical locations, and collection years from NCBI. Together with the strains isolated by us, a maximum likelihood (ML) phylogenetic tree was constructed based on the core genes using Roary v3.13.0 and FastTree v2.1.9 [56,57] and visualized through iTOL (https://itol.embl.de/).

### 2.3. Virulence Genes and ARGs Analysis

Whole-genome sequencing (WGS) was performed utilizing the Illumina NovaSeq platform. Data quality control was implemented with fastp v0.23.1. Sequence assembly was undertaken using SPAdes v3.15.4, and contigs with a length shorter than 500 bp were discarded. Annotation was carried out using Prokka 1.14.6 [58].

For ARGs analysis, the ABRicate v1.0.0 [59] was employed in conjunction with the Comprehensive Antibiotic Resistance Database (CARD, https://card.mcmaster.ca/) for alignment. The parameter settings were as follows: E-value = 1 × 10^−5^; coverage > 50%; and identity > 70%. Simultaneously, antibiotic resistance proteins within this database were also compared using local BLASTp with the following parameter settings: E-value = 1 × 10^−5^; coverage > 70%; and identity > 45%.

Regarding VF gene analysis, the ABRicate software was utilized and compared with the VFs of Pathogenic Bacteria Database (VFDB, http://www.mgc.ac.cn/VFs/) for alignment. The parameter settings were E-value = 1 × 10^−5^, coverage > 50%, and identity > 60%. Additionally, VF-related proteins in this database were compared using local BLASTp, with parameter settings of E-value = 1 × 10^−5^, coverage > 70%, and identity > 60%.

### 2.4. Growth Curve and Cytotoxicity Assay of Bacteria with T3SS2

With aseptic operation, a single colony was picked from the bacterial solid medium and inoculated into 5 mL of PNCC enrichment broth for overnight shaking culture. The concentration of the culture was adjusted to OD_600_ = 0.8, and then it was added to the 96-well plate according to a 1% inoculum concentration. Each well contained 200 μL of PNCC enrichment broth. The bacterial growth curve was established using the fully automatic microbial detector LogPhase 600 (Biotek, Clarksville, TN, USA), with the uninoculated liquid medium serving as a blank control. The temperature was set at 37 °C and the shaking speed was 500 rpm. The value at OD_600_ was read every hour for a total of 48 h (at least three parallel experiments).

Under the conditions of 37 °C, 5% CO_2_, and 95% humidity, THP-1 cells were cultured in THP-1 cell complete medium (10% FBS, 1% P/S, and 0.05 mM β-mercaptoethanol). On the day before the experiment, the cells were centrifuged and washed, and then resuspended in phenol red-free RPMI-1640 medium. Then, an appropriate number of cells were inoculated on 96-well plates and continued to be cultured for 24 h. Different bacterial suspensions (adjusted to the same concentration) were prepared to infect the cells with an MOI of 5. The cell culture supernatants were taken at 0 h, 1 h, 2 h, 3 h, 4 h, 5 h of infection, and the LDH Cytotoxicity Assay Kit (Adamas-life, Shanghai, China) was used to determine the release amount of LDH as a cytotoxicity marker (at least three parallel experiments). Different treatment groups were set up in the experiment, including experimental group A infected by bacteria, control group B not infected by bacteria, and control group C treated with lysis solution (maximum enzyme activity control). Cytotoxicity was calculated as (A − B)/(C − B) × 100%. The Synergy HTX multifunctional microplate reader (Biotek, Clarksville, TN, USA) was used to measure the absorbance at 440 nm. The absorbance was proportional to the activity of lactate dehydrogenase. And the photos of cells at different infection stages were taken by an inverted phase contrast microscope CKX53 (Olympus, Tokyo, Japan).

### 2.5. Bioinformatic Analysis of Genomes of Bacteria with T3SS2

Based on the Illumina NovaSeq sequencing platform, in conjunction with the third-generation single-molecule sequencing technology, the Oxford Nanopore ONT sequencing platform, these libraries were sequenced, respectively. The third-generation sequenced data were assembled using Unicycler v0.5.0 and Flye v2.9.1 software to obtain contig sequences. Specifically, the high-quality second-generation data were utilized to correct the third-generation contig results using pilon v1.24 software. Eventually, a complete sequence was obtained through splicing. Gene annotation was performed using Prokka 1.14.6 [58]. For genomic island (GI) prediction, the online website IslandViewer4 (https://www.pathogenomics.sfu.ca/islandviewer/browse/) was utilized. An alien hunter [60] was employed to predict the horizontal gene transfer (HGT) events. BLAST+ 2.12.0 [61] was used to conduct a BLAST comparison with other genome sequences. Finally, the online website Proksee (https://proksee.ca/) was employed for the genome circular map. The gene clusters were accomplished using the online website ChiPlot (https://www.chiplot.online/).

In the NCBI nucleotide collection (nr/nt) and whole-genome shotgun contigs (wgs) databases, BLAST was performed on the T3SS2 sequence of CNVVF081014. A total of 204 related sequences were obtained (with identity ≥ 60% and coverage ≥ 80% to CNVVF081014-T3SS2), including 20 sequences of *V. vulnificus*, 168 sequences of *V. parahaemolyticus*, 10 sequences of *V. harveyi*, 3 sequences of *V. campbellii*, 1 sequence of *V. lentus*, and 2 sequences of *Aeromonas veronii*. Simultaneously, the isolation sources, hosts, geographical locations, and collection years (as of June 2024) of these strains were retrieved. The world heatmap was drawn based on the highly related strain above.

Moreover, to further elucidate the differences of *V. vulnificus* T3SS2 among *Vibrio* spp. and the other bacterial genera, we compared CNVVF081014 with the T3SS2 sequence of other genera (with at least 50% identity and 60% coverage to CNVVF081014). Visualization was performed using the online website GBKviz (https://gbkviz.streamlit.app/). Collinearity analysis among T3SS2 sequences of different strains was conducted using Mashtree v1.0.0, and a phylogenetic tree was constructed using the neighbor-joining method of MEGA11 software [62]. Final visualization was accomplished using iTOL (https://itol.embl.de/).

### 2.6. Data Analysis

Charts and statistical analyses (Two-way ANOVA or mixed model) were generated by using GraphPad Prism 9.5 (https://www.graphpad.com/). When *p* < 0.05, there is statistical significance in the difference. The world map was generated by using the ggplot package in R Studio (https://www.r-project.org/). Both pie charts and heat maps were completed on the ChiPlot website (https://www.chiplot.online/). Image combination and layout were carried out by using Adobe Illustrator software.

## 3. Results

### 3.1. Biotypes and Genotypes of V. vulnificus from Diverse Sources

**Figure 1:**
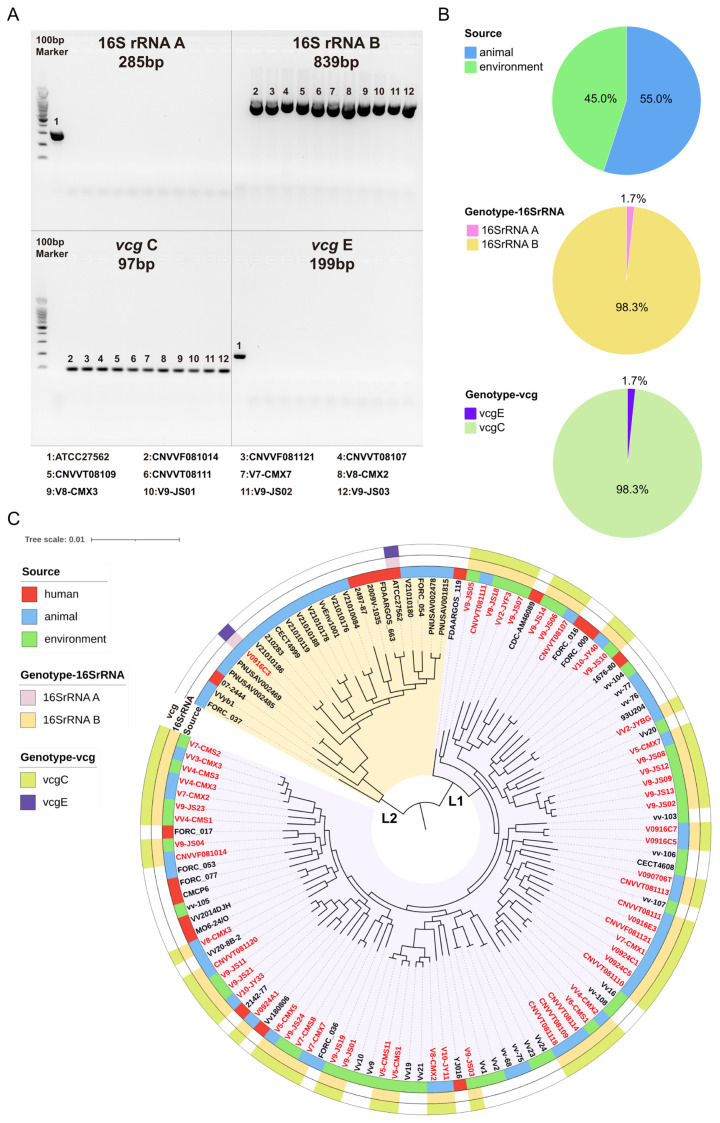
A total of 60 strains of *V. vulnificus* from environmental and zoological sources were isolated in Shanghai during the period from June to December 2023. (**A**) Genotyping of the 16S rRNA A/B and *vcg*C/E genes of *V. vulnificus* in Shanghai. (**B**) Proportions of *V. vulnificus* strains with different isolation sources, 16S rRNA A/B genotypes, and *vcg*C/E genotypes, respectively. (**C**) The maximum likelihood phylogenetic tree of *V. vulnificus* in Shanghai and those downloaded from NCBI (Strains isolated in this study are shown in red).

In this study, from June to December 2023, a total of 60 strains of *V. vulnificus* were isolated from 328 samples (18.3%) in Shanghai, China. Among these, 33 strains (55%) were obtained from animal sources, including shellfish, crabs, shrimps, and throat swabs of migratory birds, while 27 strains (45%) were sourced from the environment, such as seafood culture water and seawater. The specific locations, sources, genotypes, biotypes, and isolation times of the 60 strains are presented in the Supplementary Material (Table S1). All strains were found to carry the *vvhA* gene, and none of the isolates were of *serE* and *Bt2*. Notably, 59 strains belonged to the 16S rRNA B-*vcg*C genotype (98.3%), which was associated with higher virulence, while one strain (1.7%) exhibited the 16S rRNA A-*vcg*E genotype and was isolated from the throat swab of a migratory bird (Figure 1A,B).

Detailed information of the NCBI strains is also organized in the Supplementary Material (Table S2). Phylogenetic analysis revealed that *V. vulnificus* mainly consists of two genetic lineages (Figure 1C). A significant number of strains were clustered within Lineage 1, representing a majority of the 16S rRNA B-*vcg*C genotype strains. In contrast, only a small number of strains were identified within Lineage 2, including the ATCC27562 strain and our isolated strain V0916C3, which exhibited the 16S rRNA A-*vcg*E genotype. A notable positive correlation was observed between the 16S rRNA A/B and *vcg*C/E. Nevertheless, the correlation between genotype and strain isolation source was not particularly strong. Strains from clinical, animal, and environmental sources displayed a diverse range of genotypes and genetic lineages.

### 3.2. Antibiotic Resistance and Virulence Characteristics of V. vulnificus

**Figure 2:**
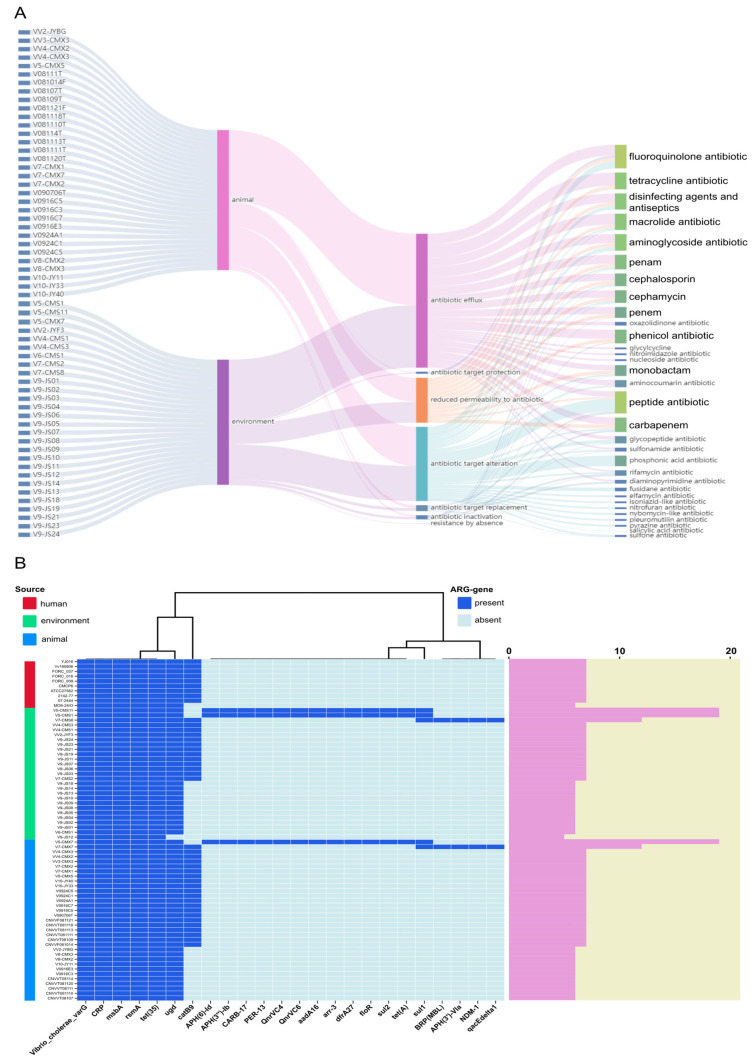
Antibiotic resistance mechanisms and ARGs associated with *V. vulnificus* strains in Shanghai. (**A**) Antibiotic resistance mechanisms and types of antibiotics involved in *V. vulnificus* from diverse sources. (**B**) ARGs harbored by different strains (Dark blue rectangles in the figure signify the presence of ARGs, while light blue rectangles indicate absence. The columns of different colors on the left represent different sources. The pink column on the right represents the total quantity of ARGs contained in different strains. To compare the differences in ARGs of *V. vulnificus* from different sources, 10 clinically sourced strains were downloaded from NCBI).

A total of 24 ARGs were identified in the genome of *V. vulnificus*. The three most prevalent resistance mechanisms were determined to be antibiotic efflux, alteration of antibiotic targets, and reduced permeability to antibiotics. In the case of the antibiotic resistance categories, the six most common ones were fluoroquinolone, peptide, tetracycline, macrolide, aminoglycoside, and penam (Figure 2A). All strains contain at least five ARGs, including *varG*, *CRP*, *msbA*, *rsmA*, and *tet(35)*, and the majority of strains also harbored *ugd* and *catB9* genes. Notably, five strains (8.3%), comprising three from environmental sources and two from animal sources, harbored over ten ARGs, thereby mediating resistance to multiple antibiotics (Figure 2B).

**Figure 3:**
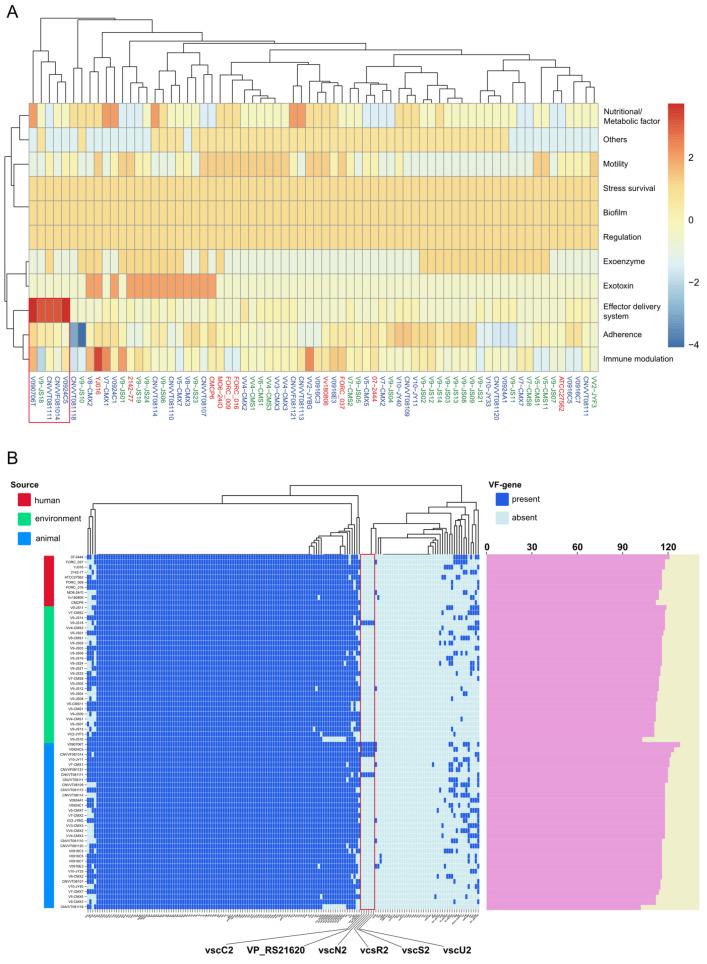
Functional classification of VFs and VF genes associated with *V. vulnificus* strains in Shanghai. (**A**) Functional categorization of VFs in *V. vulnificus* from diverse sources (Red frame: Five strains with a high content of effector delivery systems). (**B**) VF genes harbored by different strains (Dark blue rectangles in the figure signify the presence of VF genes, while light blue rectangles indicate absence. The columns of different colors on the left represent different sources. The pink column on the right represents the total quantity of VF genes contained in different strains. To compare the differences in VF genes from different sources, 10 clinically sourced strains were downloaded from NCBI; Red frame: Six genes related to T3SS2).

Concurrently, 165 VF genes were identified in the genome of *V. vulnificus*. These genes were categorized into 11 functional groups, including effector delivery systems, adherence, nutritional/metabolic factors, motility, exotoxins, immune modulation, biofilm formation, regulation, stress survival, exoenzymes, and others. No significant differences were observed in the number of VFs among the strains derived from disparate isolation sources. The principal exotoxin-related VFs identified in *V. vulnificus* were VvhA, RtxA1, TLH, hemolysin III, and hemolysin HlyA, and this bacterium had a highly abundant effector delivery system, encompassing T1SS and T2SS. It was noteworthy that five strains (one environmental source strain, V9-JS18, and four animal source strains, V0924C5, CNVVF081014, CNVVT081111, and V090706T) had a remarkably high profusion of effector delivery systems (Figure 3A). Further investigation revealed that they all uniquely contained the following six genes related to T3SS2: *vscC2*, VP_RS21620, *vscN2*, *vcsR2*, *vscS2* and *vscU2* (Figure 3B).

### 3.3. Growth and Cytotoxicity Characteristics of V. vulnificus with T3SS2

**Figure 4:**
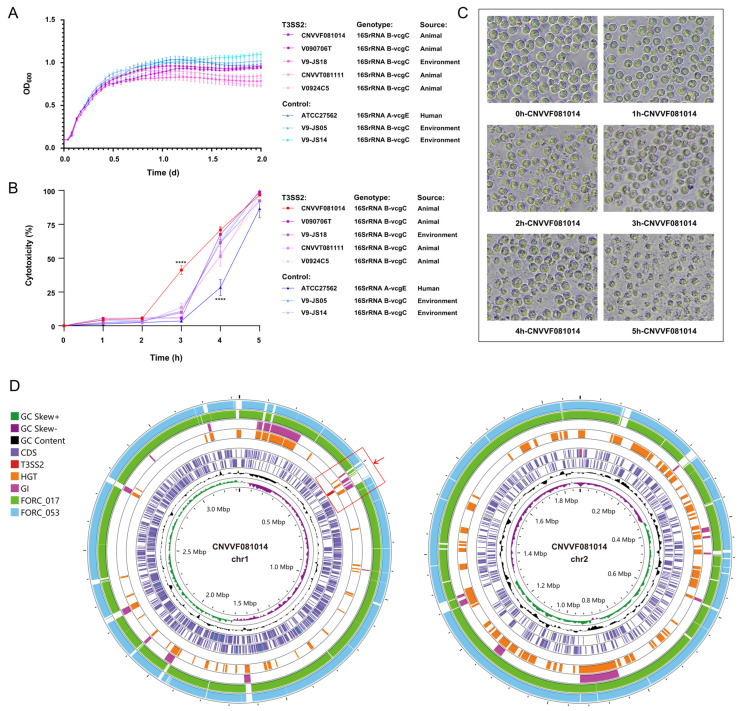
Growth curve and cytotoxicity assays of *V. vulnificus* with T3SS2; Morphological changes caused by strain CNVVF081014F infecting THP-1 and genetic map of CNVVF081014F. (**A**) In the stationary phase, the concentration of *V. vulnificus* with T3SS2 is generally lower than that of the control strain. (**B**) The cytotoxicity of *V. vulnificus* with T3SS2 is equivalent to that of the control strain or even higher in some strains (****: *p* < 0.0001). (**C**) Morphological changes in THP-1 cells caused by strain CNVVF081014F infection within 0 to 5 h. (**D**) The genetic map of strain CNVVF081014F, along with the prediction of positions of T3SS2, HGT events, and GIs on the chromosomes, and genome comparison with *V. vulnificus* strains lacking T3SS2 (Red frame: The position of T3SS2 gene cluster in strain CNVVF081014).

The bacterial growth curve indicated that all *V. vulnificus* entered the logarithmic growth phase after one hour and subsequently reached the stationary growth phase at 12 h. No significant disparity in growth rate was observed during the logarithmic phase between the strains containing T3SS2 and the control strains. However, a discrepancy became apparent after 12 h. The final OD_600_ value attained by the strains containing T3SS2 was typically lower than that of the control group, indicating that the number of strains carrying T3SS2 under the same conditions was reduced. Furthermore, no notable discrepancy was observed in the growth rate and final bacterial concentration among strains of diverse isolation sources and genotypes (Figure 4A).

The strain CNVVF081014, which contains T3SS2, induced a significant release of LDH in cells after two hours. In contrast, the cytotoxicity observed in other strains was delayed, with LDH release occurring after three hours. It was noteworthy that the 16S rRNA A-*vcg*E genotype strain ATCC27562, which served as the control group, exhibited a more pronounced delay in toxicity, with discernible LDH release occurring after four hours (Figure 4B). It was evident that not all strains containing T3SS2 exhibited a discernible increase in virulence. The 16S rRNA B-*vcg*C genotype strain had a more distinct toxic effect on cells in the early stages of infection compared to the 16S rRNA A-*vcg*E genotype strain. Morphological observations indicated that as the duration of infection increased, THP-1 cells underwent a gradual reduction in size, ultimately leading to cell rupture (Figure 4C).

The WGS data demonstrated that the CNVVF081014 strain acquired a greater number of genomic islands (GIs) transferred through horizontal gene transfer (HGT) compared to strains lacking T3SS2 (FORC_017 and FORC_053). Additionally, T3SS2 was found at the HGT locus on chromosome 1 of this strain (Figure 4D).

### 3.4. Characteristics and Differences of T3SS2 in V. vulnificus and Other Genera

We further annotated the T3SS2 gene cluster of CNVVF081014 and incorporated the 10 kb upstream and downstream regions of the annotated open reading frames (ORFs) for analysis to identify potential T3SS2 sequence signals. We identified insertion sequences (ISs), such as IS*Va8*, IS*Sba16*, IS*Vch6*, IS*Vsa5*, IS*As1*, IS*Xne3*, and IS*Vpa3*, on both sides of the upstream and downstream regions of the T3SS2 gene cluster, which corresponds to the aforementioned T3SS2 located at the HGT position in chromosome 1.

**Figure 5:**
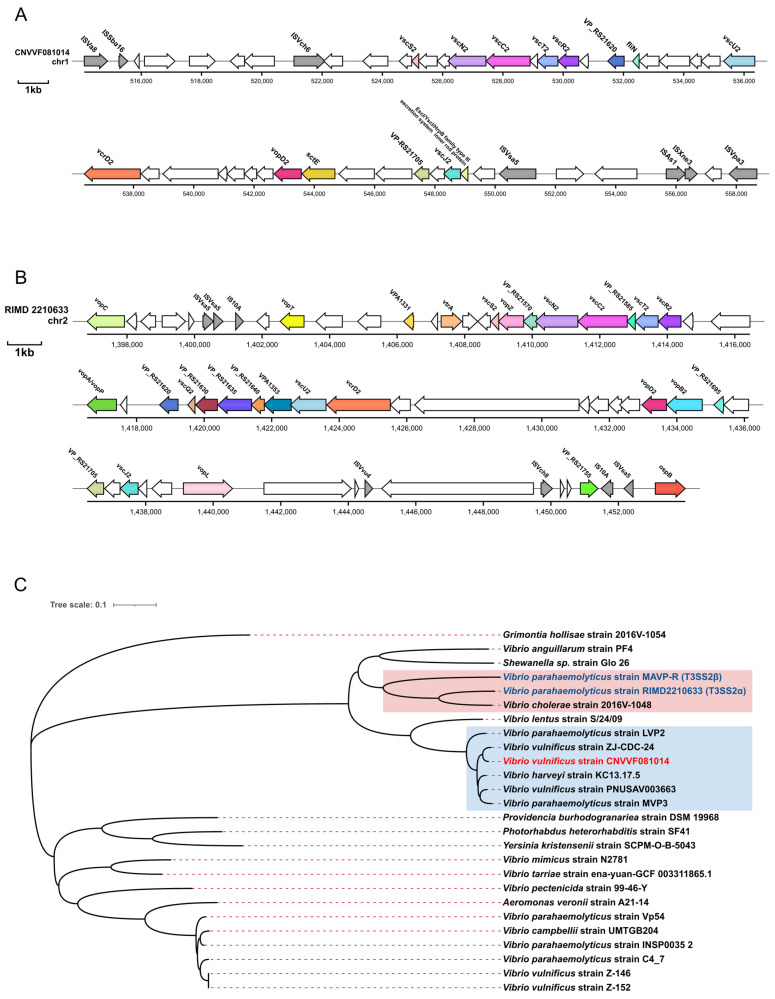
Genetic environment of T3SS2 gene cluster and NJ phylogenetic tree based on SNPs of T3SS2 gene clusters. (**A**,**B**) Genetic environment of T3SS2 of *V. vulnificus* CNVVF081014 and T3SS2α of *V. parahaemolyticus* RIMD2210633. (**C**) NJ phylogenetic tree of T3SS2 gene sequences contained in 26 bacteria of different genera (The red background represents the lineage where T3SS2α and T3SS2β are situated, and the blue background represents the lineage where CNVVF081014 is located).

T3SS2 of *V. vulnificus* contained structural protein-coding genes, including *vscN2* (SctN), *vscC2* (SctC), *vscS2* (SctS), *vscT2* (SctT), *vscR2* (SctR), *vscU2* (SctU), *vcrD2* (SctV), and *vscJ2* (SctJ); a regulatory protein-coding gene, VP_RS21620 (VtrB); and a transposon protein-coding gene, *vopD2*. Notably, compared to *V. parahaemolyticus* RIMD2210633, T3SS2 of *V. vulnificus* also possessed the flagellar motility switch protein FliN, the T3SS translocation subunit SctE, and the T3SS2 inner rod protein from the Escl/Yscl/HrpB family (Figure 5A,B).

The phylogenetic distribution analysis indicated that our strain CNVVF081014 was in the same major branch as *V. parahaemolyticus* RIMD2210633 (T3SS2α) and MAVP-R (T3SS2β) but on different minor branches. It was situated within a smaller branch alongside *V. vulnificus* ZJ-CDC-24, PNUSAV003663, *V. parahaemolyticus* LVP2, and MVP3, indicating that *V. vulnificus* T3SS2 represented a disparate lineage from T3SS2α and T3SS2β. The T3SS2 of other bacterial genera, including *Aeromonas*, *Providencia*, *Photorhabdus*, *Yersinia*, and *Grimontia*, were situated in more distant branches (Figure 5C).

**Figure 6:**
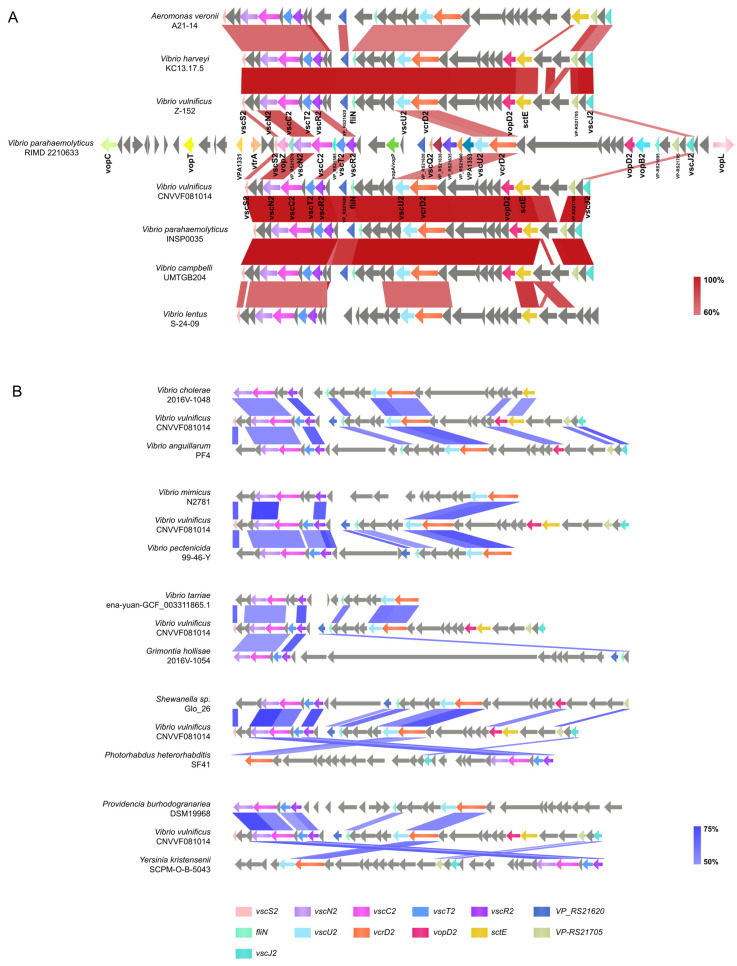
Comparative study of T3SS2 gene clusters. (**A**) Comparison of bacterial gene clusters highly similar (identity ≥ 60%, coverage ≥ 80%) to T3SS2 of *V. vulnificus* CNVVF081014. (**B**) Comparison of bacterial gene clusters less similar (identity ≥ 50%, coverage ≥ 60%) to T3SS2 of *V. vulnificus* CNVVF081014 (Arrows indicate the position and direction of genes: T3SS2 functional genes are marked with colored legends, and other genes are represented in gray).

T3SS2α exhibited a higher degree of internal gene richness, containing a greater number of effector proteins than the internal gene cluster of *V. vulnificus*, such as VopC, VopT, VopZ, VopA/VopP, and VopL. *V. vulnificus* T3SS2 demonstrated over 90% similarity with some other bacteria of the *Vibrio* genus, such as *V. parahaemolyticus* INSP0035, *V. campbelli* UMTGB204, and *V. harveyi* KC13.17.5 (Figure 6A). In contrast, compared to other bacterial genera, the similarity within the gene cluster was even lower, only manifesting similarities in some T3SS2 structural proteins such as SctN, SctC, SctT, SctR, SctU, and SctV (Figure 6B).

### 3.5. Geographical Distribution of Strains with Sequences Similar to V. vulnificus T3SS2

**Figure 7:**
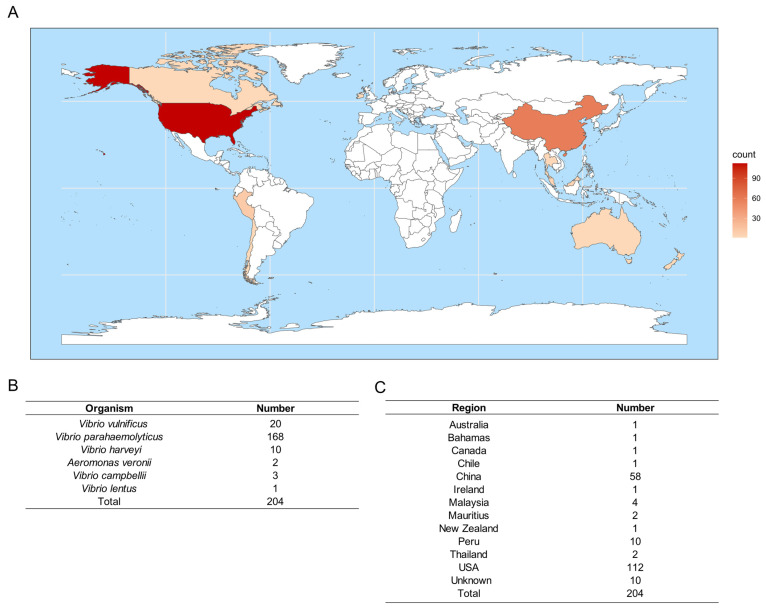
Worldwide distribution of strains containing sequences highly similar to T3SS2 of *V. vulnificus*. (**A**) Heat map of worldwide distribution of bacteria that are highly similar (identity ≥ 60%, coverage ≥ 80%) to T3SS2 of *V. vulnificus* CNVVF081014. (**B**,**C**) Bacterial species and regions of these strains with high similarity.

To gain insight into the global geographical distribution of strains exhibiting high similarity to the T3SS2 gene cluster of *V. vulnificus*, we constructed a world heatmap of these strains (Figure 7A). As of June 2024, a total of 204 related sequences were retrieved using the T3SS2 sequence of CNVVF081014 as a reference, with an identity of ≥60% and coverage of ≥80%. The largest proportion of the retrieved sequences was that of *V. parahaemolyticus*, constituting 168 sequences (82.3%) of the total. This was followed by 20 sequences (9.8%) of *V. vulnificus*, 10 sequences (4.9%) of *V. harveyi*, 3 sequences (1.5%) of *V. campbellii*, 2 sequences (1.0%) of *A. veronii*, and 1 sequence (0.5%) of *V. lentus* (Figure 7B). Among these, 112 strains (54.9%) were from the United States, representing the highest abundance, followed by 58 strains (28.4%) identified as originating from China (Figure 7C). It was evident that a majority of the strains were concentrated in coastal countries, a consequence of the high prevalence of *Vibrio* bacteria in these regions, which facilitated the dissemination of the T3SS2 gene cluster.

## 4. Discussion

Infection with *V. vulnificus* is potentially fatal [63,64], which underscores the necessity of investigating antibiotic resistance and virulence traits to effectively control and prevent its spread. We analyzed the molecular typing of 60 strains isolated from the environment and animals in Shanghai, China. Our findings revealed that all isolates lacked the *SerE* gene and did not belong to biotype 2 [13]. Given that biotype 3 was a chimera of biotype 1 and biotype 2 and was restricted in distribution to Israel [10], it could be speculated that all these strains belonged to biotype 1. Notably, the predominant genotypes identified in our research were 16S rRNA B-*vcg*C (98.3%, 59/60), which were considered to possess relatively high virulence potential [21]. Furthermore, we confirmed that 16S rRNA A-*vcg*E (EA) were mainly located in Lineage 2, whereas 16S rRNA B-*vcg*C (CB) were predominantly found in Lineage 1. Although both genotypes may express virulence in mouse models, *vcg*C-positive strains are more likely to be associated with systemic infection and mortality [65]. In Ningbo, 21 strains of *V. vulnificus* from clinical sources were of the *vcg*C genotype, with a significant proportion (16 out of 21) identified as 16S rRNA B [66]. Our study contrasts with the prevailing view that most strains isolated from the environment are EA genotype strains [21,67,68]. In addition, all 105 strains of *V. vulnificus* isolated from seafood and the environment in Guangzhou (south of China) are also CB genotypes (unpublished results from our group). Therefore, we conclude that there is no direct correlation between the genotype and the source of isolation. A significant number of CB genotype strains are present in environmental sources, extending beyond clinical sources. Interestingly, a study conducted in the United States revealed that phylogenetic lineages could not reliably predict the severity of clinical infections; the authors suggested that *V. vulnificus* from two major phylogenetic lineages could both lead to severe human diseases [69]. Roig performed an in-depth phylogenetic analysis, indicating that different lineages share virulence genes associated with human pathogenicity [25]. This suggests that all strains of this species may pose a risk to human health. Consequently, we advocate for enhanced environmental monitoring of all *V. vulnificus* strains, rather than restricting it solely to CB genotype strains.

Previous antimicrobial susceptibility testing (AST) studies on *V. vulnificus* have revealed a significant increase in resistance to antibiotics [70,71]. In a study conducted in the United States, it was observed that 45% of environmental isolates were resistant to three or more antibiotics, while 17.3% were resistant to eight or more antibiotics, including doxycycline, tetracycline, aminoglycosides, and cephalosporins, which were typically employed in the treatment of infections [72]. Similarly, a recent study indicated that the majority of *V. vulnificus* strains derived from clinical sources were multidrug-resistant, accounting for 66.7%. These strains exhibited resistance to imipenem (100%), vancomycin (80.95%), cefradine (66.67%), polymyxin B (61.90%), and streptomycin (47.62%) [66]. In our research, the ARGs identified in most strains included *varG*, *CRP*, *msbA*, *rsmA*, *tet(35)*, *ugd*, and *catB9*. These genes conferred resistance to a range of antibiotics, such as penicillins, carbapenems, cephalosporins, fluoroquinolones, peptides, tetracyclines, macrolides, aminoglycosides, etc., thereby elucidating some of the previously observed antibiotic resistance phenotypes. While some studies indicate that first-line antibiotics used to treat severe vibriosis remain effective [73,74], strains from other regions demonstrate the emergence of new antibiotic-resistant phenotypes [66,75,76]. This may be linked to the extensive use of antibiotics in intensive aquaculture. Research findings indicated that the incorporation of antibiotics into fish feed contributed to the rising prevalence of resistance genes for macrolides, tetracyclines, aminoglycosides, and chloramphenicol in underwater sediments [77]. Additional studies demonstrated that antibiotics used in aquaculture were continuously discharged into the marine environment via rivers, with several tons of antibiotics being transported to the coastal waters annually [78,79,80], which increased the degree of bacterial resistance in the natural environment. These findings underscore the necessity for continued prudent antibiotic use in both clinical settings and aquaculture to mitigate the worsening of treatment challenges.

In our study, 60 strains exhibited a significant abundance of VF genes, which may predominantly contribute to the high fatality rate associated with *V. vulnificus* infections. These isolates possessed a range of VF genes, including those linked to acid neutralization, capsular polysaccharide, iron acquisition, motility, and adhesion. Several genes encoded exotoxins (such as VvhA, RtxA1, etc.) and secretion systems (T1SS and T2SS). Notably, five strains uniquely contained T3SS2-related genes, which have not been reported in prior studies. The T3SS is a “molecular syringe” that directly introduces or injects bacterial toxins into the cytoplasm of host cells [81]. This secretion system was first identified in *V. parahaemolyticus* and contained two T3SSs—T3SS1 and T3SS2 [40]. Animal infection model studies manifested that T3SS1 played a minor role in the infection process, whereas T3SS2 was crucial for enterotoxicity and the manifestation of clinical symptoms [82,83]. T3SS2 implicated in the pandemic associated with a *V. parahaemolyticus* O3:K6 clone [51]. Furthermore, in a study of non-O1/O139 *V. cholerae* strains isolated in Chile, it was found that most strains (6/9) contained T3SS2-related genes located on chromosomal pathogenicity islands [84]. Given that these pathogenic islands could be partially or completely transferred [85], the threat posed by non-O1/O139 *V. cholerae* to human health was heightened. Based on the above findings, we hypothesize that the *V. vulnificus* strains with T3SS2 in this study likewise possess such potential, posing a greater threat to human health compared to common strains.

Cytotoxicity assays revealed that strain CNVVF081014 exhibited significant toxicity to cells at the early stage (2 h) compared to other strains. Nevertheless, not all strains containing T3SS2 displayed obvious virulence at the second hour; most displayed virulence after the third hour. This variation can be attributed to the highly sophisticated and intricate virulence system of *V. vulnificus* [33,86]. The interaction between bacteria and cells also mediates cytotoxicity through RtxA1 [87,88]. Furthermore, the CB genotype strains were observed to exert a more pronounced toxic effect on cells in the early stages of infection compared to the EA genotype strains. This finding aligns with the established understanding of the virulence differences among the various genotypes of *V. vulnificus* [22,23]. Although the EA genotype strain initially displayed a lag in virulence, it ultimately reached a comparable cell lethality rate to that of the CB genotype strains, suggesting that the virulence of the EA genotype strain should not be underestimated.

Our analysis uncovered multiple GIs in the genome of CNVVF081014, with T3SS2 located on a GI in chromosome 1. This finding is consistent with previous observations that genes encoding T3SS and effectors are often horizontally acquired by the bacteria that utilize them [89]. These genes are typically found on chromosomal pathogenicity islands or, less frequently, on virulence-related plasmids. The mutual transfer of pathogenicity islands facilitates the acquisition of T3SS2 among different bacterial species. Furthermore, in conjunction with the growth curve results, we hypothesize that multiple HGT events also contributed to an increased copy number of the bacterial genome, potentially accounting for the observed differences in bacterial quantity during the later stages of growth.

The genetic environment analysis of *V. vulnificus* T3SS2 revealed the presence of numerous ISs within 10 kilobases upstream and downstream of the gene cluster. The genes encoding transposases further confirmed the transferability of T3SS2 gene cluster. Since a majority of our understanding of T3SS2 has been derived from studies of T3SS2α in the bacterial species *V. parahaemolyticus* RIMD2210633, it follows that, compared to the classic T3SS2α, both *V. parahaemolyticus* and *V. vulnificus* contain the structural proteins SctN/C/S/T/R/U/V/J, the regulatory protein VtrB, and the translocon protein VopD2. Notably, *vscN2* (SctN) and *vscC2* (SctC) encode two of the most conserved T3SS structural proteins, which are frequently employed for the phylogenetic clustering and classification of T3SS [90,91]. SctN is a component of the ATPase complex, while SctC is part of the output device. Importantly, the T3SS2 of *V. vulnificus* uniquely had a flagellar motor switch protein FliN, the T3SS translocon subunit SctE, and T3SS2 inner rod protein from the Escl/Yscl/HrpB family. The presence of flagellar-related genes can be attributed to the relationship between T3SS and bacterial flagella [89]. Studies have indicated that T3SS originated from a loss of the flagella, wherein portions of the flagellar structures were recruited for the evolution of new protein transfer functions [91]. And the assembly pathway of T3SS is similar to that of the flagella, including the switching of secreted substrates during appendage formation [92,93] and inside–out assembly process [94]. In addition, the comparison between gene clusters indicated that the gene abundance of *V. parahaemolyticus* T3SS2α exceeded that of *V. vulnificus*. This observation can be attributed to the former’s possession of a diverse array of type III secreted effectors (T3SEs), including VopC, VopT, VopZ, VopA/VopP, and VopL. Currently, 15 distinct T3SEs have been identified [51,95]. Among these, five are classified as core effector proteins (VopZ, VopV, VopG, VopL, and VgpA) due to their presence in both *V. parahaemolyticus* and *V. cholerae*. The remaining ten are categorized as accessory effector proteins. The difference in effector proteins also has also placed different bacterial genera in different lineages in phylogenetic analysis of T3SS2. Studies have shown that genes encoding bacterial effector proteins can be acquired through HGT or can evolve further via terminal reassortment and pseudogenization events [89,96,97]. This supports the view that different bacterial species will select different effector proteins based on their adaptation to different niches. Consequently, T3SS2-positive strains may use different repertoires of effector proteins to interact with environmental eukaryotic hosts in varied ways, ultimately affecting their pathogenic potential. The T3SEs of *V. vulnificus* remain incompletely characterized. We propose that future investigations should prioritize the identification of T3SS2 effector proteins in *V. vulnificus* and examine their roles in bacterial pathogenicity and environmental adaptation.

Compared to other bacterial genera, the internal differences of T3SS2 are particularly pronounced. Notably, with the exception of structural proteins such as SctN/C/T/R/U/V, the degree of similarity among the remaining genes was relatively low. This disparity arises from the fact that, despite certain shared characteristics, T3SEs exhibit significant evolutionary diversification [96]. They demonstrate a high degree of variation both within and between species [98]. The close interactions between the T3SEs and various hosts expose them to intense selection pressures [99], resulting in rapid evolutionary changes [100]. Contrary to the previous reports that emphasize the introduction of genetic variation through intragenic homologous recombination [101,102], the evolution of T3SEs is significantly influenced by non-homologous recombination processes. This phenomenon not only accounts for the high frequency of truncated and chimeric T3SEs but also provides insights into how the unique structure of these genes fosters dramatic evolutionary and functional changes [103]. Such changes may play a crucial role in the ongoing arms race between pathogens and hosts. In terms of global geographical distribution, *Vibrio* species with sequences similar to T3SS2 of CNVVF081014 are predominant, particularly in coastal countries. This prevalence can be attributed to the fact that *Vibrio* spp. thrives abundantly in coastal regions, which may facilitate the dissemination of the T3SS2 gene cluster. Several studies have unveiled that, on account of the influences of elevated water temperatures [104] and adverse weather conditions associated with climate change, the hazard of being infected by *V. vulnificus* in coastal regions has increased [105,106,107]. The detection of *V. vulnificus* harboring T3SS2, as a novel threat, undoubtedly adds insult to injury to this already precarious circumstance.

## 5. Conclusions

This study revealed that a majority of the *V. vulnificus* strains (98.3%, 59/60) isolated from the environment and animals in Shanghai belonged to the 16S rRNA B-*vcg*C genotype, which undoubtedly increases the risk of human infection. All strains possessed ARGs including *varG*, *CRP*, *msbA*, *rsmA*, and *tet(35)*, which mediate resistance to multiple antibiotics. The presence of 165 virulence genes endowed *V. vulnificus* with high virulence. Additionally, the newly discovered T3SS2 further enhanced virulence in several strains. The transferability of the T3SS2 gene cluster in *V. vulnificus* has been demonstrated, and the transfer of pathogenicity islands between *Vibrio* strains facilitated this acquisition. Furthermore, the dissimilarities in the T3SEs of *V. vulnificus* positioned it within a disparate T3SS2 evolutionary lineage, separate from that of *V. parahaemolyticus*. Cytotoxicity experiments confirmed that some strains harboring T3SS2 exhibited early virulence release, representing a new threat to humans. The risk of infection with *V. vulnificus* in coastal areas is still increasing. Therefore, it is imperative to continuously strengthen comprehensive environmental monitoring to achieve early detection and timely intervention.

## Data Availability

The whole genome sequence data reported in this paper have been deposited in the Genome Warehouse in National Genomics Data Center, Beijing Institute of Genomics, Chinese Academy of Sciences/China National Center for Bioinformation, under BioProject PRJCA025669 and PRJCA030422, that are publicly accessible at https://ngdc.cncb.ac.cn/gwh.

## Supplementary Materials

The following supporting information can be downloaded at: https://www.mdpi.com/article/10.3390/microorganisms12112375/s1, Table S1: Information concerning *V. vulnificus* strains isolated from shanghai, China. Table S2: Information concerning *V. vulnificus* strains from the NCBI database.
