# Supplementary material for: Genomic Insight into Zoonotic and Environmental Vibrio vulnificus: Strains with T3SS2 as a Novel Threat to Public Health
Source: Microorganisms. 2024 Nov 20;12(11):2375. doi: 10.3390/microorganisms12112375 (PMC11596471; doi:10.3390/microorganisms12112375)
Supplement: Supplementary file 1 [file microorganisms-12-02375-s001.zip › Supplementary tables.pdf]

Table S1. Information concerning *V. vulnificus* strains isolated from shanghai, China.

Strain	Location	Source	Genotype-vcg	Genotype-16SrRNA	Biotype	Collection date
VV4-CMS3	China:shanghai	environment(Shrimp culture water)	C	B	BT1	2023
VV4-CMS1	China:shanghai	environment(Shrimp culture water)	C	B	BT1	2023
VV2-JYF3	China:shanghai	environment(Shrimp culture water)	C	B	BT1	2023
V7-CMS8	China:shanghai	environment(Shrimp culture water)	C	B	BT1	2023
V7-CMS2	China:shanghai	environment(Shrimp culture water)	C	B	BT1	2023
V6-CMS1	China:shanghai	environment(Shrimp culture water)	C	B	BT1	2023
V5-CMS11	China:shanghai	environment(Shrimp culture water)	C	B	BT1	2023
V5-CMS1	China:shanghai	environment(Shrimp culture water)	C	B	BT1	2023
V9-JS24	China:shanghai	environment(seawater)	C	B	BT1	2023
V9-JS23	China:shanghai	environment(seawater)	C	B	BT1	2023
V9-JS21	China:shanghai	environment(seawater)	C	B	BT1	2023
V9-JS19	China:shanghai	environment(seawater)	C	B	BT1	2023
V9-JS18	China:shanghai	environment(seawater)	C	B	BT1	2023
V9-JS14	China:shanghai	environment(seawater)	C	B	BT1	2023
V9-JS13	China:shanghai	environment(seawater)	C	B	BT1	2023
V9-JS12	China:shanghai	environment(seawater)	C	B	BT1	2023
V9-JS11	China:shanghai	environment(seawater)	C	B	BT1	2023
V9-JS10	China:shanghai	environment(seawater)	C	B	BT1	2023
V9-JS09	China:shanghai	environment(seawater)	C	B	BT1	2023
V9-JS08	China:shanghai	environment(seawater)	C	B	BT1	2023
V9-JS07	China:shanghai	environment(seawater)	C	B	BT1	2023
V9-JS06	China:shanghai	environment(seawater)	C	B	BT1	2023
V9-JS05	China:shanghai	environment(seawater)	C	B	BT1	2023
V9-JS04	China:shanghai	environment(seawater)	C	B	BT1	2023
V9-JS03	China:shanghai	environment(seawater)	C	B	BT1	2023
V9-JS02	China:shanghai	environment(seawater)	C	B	BT1	2023
V9-JS01	China:shanghai	environment(seawater)	C	B	BT1	2023
V0924C5	China:shanghai	animal(wildbird)	C	B	BT1	2023
V0924C1	China:shanghai	animal(wildbird)	C	B	BT1	2023
V0924A1	China:shanghai	animal(wildbird)	C	B	BT1	2023
V0916E3	China:shanghai	animal(wildbird)	C	B	BT1	2023
V0916C7	China:shanghai	animal(wildbird)	C	B	BT1	2023
V0916C5	China:shanghai	animal(wildbird)	C	B	BT1	2023
V0916C3	China:shanghai	animal(wildbird)	E	A	BT1	2023
V090706T	China:shanghai	animal(wildbird)	C	B	BT1	2023
CNVVT08114	China:shanghai	animal(wildbird)	C	B	BT1	2023
CNVVT081120	China:shanghai	animal(wildbird)	C	B	BT1	2023
CNVVT081118	China:shanghai	animal(wildbird)	C	B	BT1	2023
CNVVT081113	China:shanghai	animal(wildbird)	C	B	BT1	2023
CNVVT081111	China:shanghai	animal(wildbird)	C	B	BT1	2023

CNVVT081110	China:shanghai	animal(wildbird)	C	B	BT1	2023
CNVVT08111	China:shanghai	animal(wildbird)	C	B	BT1	2023
CNVVT08109	China:shanghai	animal(wildbird)	C	B	BT1	2023
CNVVT08107	China:shanghai	animal(wildbird)	C	B	BT1	2023
CNVVF081121	China:shanghai	animal(wildbird)	C	B	BT1	2023
CNVVF081014	China:shanghai	animal(wildbird)	C	B	BT1	2023
VV4-CMX3	China:shanghai	animal(seafood)	C	B	BT1	2023
VV4-CMX2	China:shanghai	animal(seafood)	C	B	BT1	2023
VV3-CMX3	China:shanghai	animal(seafood)	C	B	BT1	2023
VV2-JYBG	China:shanghai	animal(seafood)	C	B	BT1	2023
V8-CMX3	China:shanghai	animal(seafood)	C	B	BT1	2023
V8-CMX2	China:shanghai	animal(seafood)	C	B	BT1	2023
V7-CMX7	China:shanghai	animal(seafood)	C	B	BT1	2023
V7-CMX2	China:shanghai	animal(seafood)	C	B	BT1	2023
V7-CMX1	China:shanghai	animal(seafood)	C	B	BT1	2023
V5-CMX7	China:shanghai	animal(seafood)	C	B	BT1	2023
V5-CMX5	China:shanghai	animal(seafood)	C	B	BT1	2023
V10-JY40	China:shanghai	animal(seafood)	C	B	BT1	2023
V10-JY33	China:shanghai	animal(seafood)	C	B	BT1	2023
V10-JY11	China:shanghai	animal(seafood)	C	B	BT1	2023

Table S2. Information concerning *V. vulnificus* strains from the NCBI database.

Strain	Assembly	Level	Location	Isolation source/type	Collection date
VvEnv1001	GCA_003047125.1	Complete Genome	USA: Louisiana	Oyster/Environment	Unknown
CECT4999	GCA_002215135.1	Complete Genome	Spain	Anguilla anguilla	1990
VVyb1	GCA_000342305.2	Chromosome	Israel	tilapia fish	2004
93U204	GCA_000746665.1	Complete Genome	Taiwan: Kaohsiung	Oreochromis	2004
FORC_036	GCA_002117205.1	Complete Genome	South Korea	Mactra veneriformis	Unknown
FORC_037	GCA_002204915.1	Complete Genome	South Korea	environmental/other	Unknown
FORC_054	GCA_002863725.1	Complete Genome	South Korea	Konosirus punctatus	2014/12/24
FORC_053	GCA_003522555.1	Complete Genome	South Korea	Mactra veneriformis	2013/11/13
VV20-8B-2	GCA_025994915.1	Complete Genome	Japan:Kanagawa	homogenised shrimp	2020/2/3
vv-75	GCA_015777845.1	Contig	China:Beijing	razor clam	Sep-16
vv-68	GCA_015777605.1	Contig	China:Beijing	razor clam	Sep-16
vv-77	GCA_015776235.1	Contig	China:Beijing	clam	Sep-16
vv-76	GCA_015776195.1	Contig	China:Beijing	shrimp	Sep-16
210283	GCA_017637485.1	Contig	USA:NJ	oyster	2018/7/30
V21010186	GCA_022636735.1	Contig	USA:NJ	oyster	2018/7/30
V21010084	GCA_021764005.1	Contig	USA:NJ	oyster	2021/6/28
V21010176	GCA_022609905.1	Contig	USA:NJ	oyster	2017/6/19
V21010119	GCA_022020455.1	Contig	USA:NJ	oyster	2021/7/26
V21010178	GCA_022611995.1	Contig	USA:NJ	oyster	2018/7/9

V21010188	GCA_022881795.1	Contig	USA:NJ	oyster	2018/8/8
V21010180	GCA_022609925.1	Contig	USA:NJ	oyster	2018/7/9
PNUSAV001815	GCA_019442565.1	Contig	USA	Oyster	2021/7/20
PNUSAV002478	GCA_021715395.1	Contig	USA	Oyster	2022/1/10
PNUSAV002469	GCA_021715645.1	Contig	USA	Oyster	2022/1/10
PNUSAV002485	GCA_021716775.1	Contig	USA	Oyster	2022/1/10
Vv2	GCA_017291275.1	Contig	China:Shenzhen	environmental	2016/9/18
Vv1	GCA_017291335.1	Contig	China:Shenzhen	environmental	2016/9/18
vv-108	GCA_015776895.1	Contig	China:Guangdong	pearl river	2018/3/21
Vv16	GCA_017291195.1	Contig	China:Shenzhen	environmental	2016/9/26
Vv20	GCA_017291155.1	Contig	China:Shenzhen	environmental	2016/10/17
Vv21	GCA_017291075.1	Contig	China:Shenzhen	environmental	2016/10/17
Vv19	GCA_017291205.1	Contig	China:Shenzhen	environmental	2016/9/26
Vv10	GCA_017291255.1	Contig	China:Shenzhen	environmental	2016/9/26
Vv9	GCA_017291305.1	Contig	China:Shenzhen	environmental	2016/9/18
Vv23	GCA_017290755.1	Contig	China:Shenzhen	environmental	2016/10/17
Vv24	GCA_017291135.1	Contig	China:Shenzhen	environmental	2016/10/17
vv-107	GCA_016109635.1	Contig	China:Guangdong	pearl river	2018/3/21
vv-106	GCA_016576055.1	Contig	China:Guangdong	pearl river	2018/3/21
vv-105	GCA_015776915.1	Contig	China:Guangdong	pearl river	2018/3/21
vv-104	GCA_015776755.1	Contig	China:Guangdong	pearl river	2018/1/19
vv-103	GCA_015776655.1	Contig	China:Guangdong	pearl river	2018/1/19
CECT4608	GCA_002903785.1	Contig	Spain	Eel tank water	1990
07-2444	GCA_009764115.1	Complete Genome	USA: Illinois	blood	2007/9/21
2009V-1035	GCA_009665455.1	Chromosome	USA	clinical	Unknown
CDC-AM46089	GCA_025659485.1	Contig	USA: Tennessee	acquired via water ingestion, lead to skin infection	2010
FDAARGOS_663	GCA_008693685.1	Complete Genome	USA: KY	clinical isolate	Unknown
FORC_009	GCA_001433435.1	Complete Genome	South Korea: Masan	stool sample	2008
FORC_016	GCA_001653775.1	Complete Genome	South Korea: Jeollabuk-do	blood sample	2009/7/31
YJ016	GCA_000009745.1	Complete Genome	Unknown	Unknown	Unknown
MO6-24/O	GCA_000186585.1	Complete Genome	Unknown	Unknown	1986
FDAARGOS_119	GCA_001558515.2	Complete Genome	USA	human	Unknown
FORC_017	GCA_001675245.1	Complete Genome	South Korea: Jeju island	blood sample	2014/9/18
VV2014DJH	GCA_002850455.1	Complete Genome	China: Taizhou	blood	2014/7/17
FORC_077	GCA_004319645.1	Complete Genome	South Korea	human	2017
CMCP6	GCA_004355205.1	Contig	Korea	Chonnam National University Hospital	2003,Sep-16
2142-77	GCA_009665475.1	Complete Genome	USA	clinical	Unknown
1676-80	GCA_012275045.1	Chromosome	USA	water	Unknown
Vv180806	GCA_014107515.1	Complete Genome	China: Guangzhou	blood	2018/8/6
2497-87	GCA_014211935.1	Complete Genome	USA	blood	Unknown
ATCC27562	GCA_002224265.1	Contig	USA	marine	1979
